# Supplementary material for: Species and cell-type properties of classically defined human and rodent neurons and glia
Source: eLife. 2018 Oct 15;7:e37551. doi: 10.7554/eLife.37551 (PMC6188473; doi:10.7554/eLife.37551)
Supplement: Transparent reporting form [file elife-37551-transrepform.docx]

***eLife’s* transparent reporting form**

We encourage authors to provide detailed information *within their submission* to facilitate the interpretation and replication of experiments. Authors can upload supporting documentation to indicate the use of appropriate reporting guidelines for health-related research (see [EQUATOR Network](http://www.equator-network.org/%20)), life science research (see the [BioSharing Information Resource](https://biosharing.org/)), or the [ARRIVE guidelines](http://www.plosbiology.org/article/info:doi/10.1371/journal.pbio.1000412) for reporting work involving animal research. Where applicable, authors should refer to any relevant reporting standards documents in this form.

If you have any questions, please consult our Journal Policies and/or contact us: [editorial@elifesciences.org](mailto:editorial@elifesciences.org).

**Sample-size estimation**

- You should state whether an appropriate sample size was computed when the study was being designed
- You should state the statistical method of sample size computation and any required assumptions
- If no explicit power analysis was used, you should describe how you decided what sample (replicate) size (number) to use

Please outline where this information can be found within the submission (e.g., sections or figure legends), or explain why this information doesn’t apply to your submission:

No power calculations were performed but the sample sizes used in this study are comparable to previous studies featuring next-generation sequencing data. Detailed information including replicate information for all samples can be found in Table S1, but in general, 2-4 biological replicates were generated for most sample types. Since the correlation values for replicates (Figure 1F, 2D) were high, we found that we had sufficient power to compare samples, especially since we were most interested in finding large rather than subtle changes in gene expression (e.g. between cell types or across species).

For studies of variation across human individuals, we designed our study so that we could examine the effects of age and gender on gene expression. We obtained samples from 4 individuals for 4 age ranges (20-30, 40-50, 60-70, 80-90 years), roughly equally split between males and females (7 males, 9 females). From these 16 samples we sorted for and generated gene expression profiles for 3 different cerebellar cell types (granule, basket, glia). Two glial samples were found to have granule cell contamination post-hoc and were excluded from further analysis.

**Replicates**

- You should report how often each experiment was performed
- You should include a definition of biological versus technical replication
- The data obtained should be provided and sufficient information should be provided to indicate the number of independent biological and/or technical replicates
- If you encountered any outliers, you should describe how these were handled
- Criteria for exclusion/inclusion of data should be clearly stated
- High-throughput sequence data should be uploaded before submission, with a private link for reviewers provided (these are available from both GEO and ArrayExpress)

Please outline where this information can be found within the submission (e.g., sections or figure legends), or explain why this information doesn’t apply to your submission:

Detailed information about all datasets generated, including information about replicates, can be found in Table S1. Only biological replicates (independent animals or donors) were used in this study. High throughput sequence data have been deposited in GEO under accession GSE101918 and can be accessed by reviewers using the secure token avojmmgwzpkzfov.

**Statistical reporting**

- Statistical analysis methods should be described and justified
- Raw data should be presented in figures whenever informative to do so (typically when N per group is less than 10)
- For each experiment, you should identify the statistical tests used, exact values of N, definitions of center, methods of multiple test correction, and dispersion and precision measures (e.g., mean, median, SD, SEM, confidence intervals; and, for the major substantive results, a measure of effect size (e.g., Pearson's r, Cohen's d)
- Report exact p-values wherever possible alongside the summary statistics and 95% confidence intervals. These should be reported for all key questions and not only when the p-value is less than 0.05.

Please outline where this information can be found within the submission (e.g., sections or figure legends), or explain why this information doesn’t apply to your submission:

For RNA-seq studies, statistical methods can be found in Methods section Gene count normalization, differential expression, and principle components analysis, as well as in the text for each analysis. In general, we used the R package DESeq2 to perform differential expression analysis and used an adjusted p-value cutoff of 0.01 with no fold change cutoff. Any deviation is described in the text describing the analysis. For example, for the mouse-human comparative studies, in order to focus on large changes that are unlikely to be due to annotation artifacts, we used very stringent statistical and filtering strategy, which is detailed in the text as well as Figure S5. Full output from the differential expression analyses can be found in Table S4 and S5.

For the ATAC-seq study, details about statistics can be found in the legend for Figure 4.

Statistics for GO analysis can be found in Methods under section GO analysis. Full results of GO analyses can be found in Table S6.

(For large datasets, or papers with a very large number of statistical tests, you may upload a single table file with tests, Ns, etc., with reference to sections in the manuscript.)

**Group allocation**

- Indicate how samples were allocated into experimental groups (in the case of clinical studies, please specify allocation to treatment method); if randomization was used, please also state if restricted randomization was applied
- Indicate if masking was used during group allocation, data collection and/or data analysis

Please outline where this information can be found within the submission (e.g., sections or figure legends), or explain why this information doesn’t apply to your submission:

Samples were group based on species and cell type. For human studies, samples were group based on clinical data such as gender, age, and autolysis time (post-mortem delay). Post-hoc analysis revealed a group of 3 individuals that had a gene expression signature that differed from other individuals. Two out of 16 human glial samples were found to have granule cell contamination and were excluded from further analysis.

**Additional data files (“source data”)**

- We encourage you to upload relevant additional data files, such as numerical data that are represented as a graph in a figure, or as a summary table
- Where provided, these should be in the most useful format, and they can be uploaded as “Source data” files linked to a main figure or table
- Include model definition files including the full list of parameters used
- Include code used for data analysis (e.g., R, MatLab)
- Avoid stating that data files are “available upon request”

Please indicate the figures or tables for which source data files have been provided:

Raw (fastq) and processed (counts) sequencing data have been deposited in GEO. R scripts used to perform the analysis and required annotation files for these scripts have been deposited into the Github repository: <https://github.com/xu-xiao/paper-non-transgenic-cell-type-profiling>
